# Identifying flaws in the GWAS datasets of a published Mendelian randomization study: complementary re-evaluation and suggestion for analytical refinements

**DOI:** 10.1186/s12967-024-05106-w

**Published:** 2024-03-26

**Authors:** Jia-Cheng Xiang, Yi-Fan Xiong, Shao-Gang Wang, Qi-Dong Xia

**Affiliations:** Department and Institute of Urology, Tongji Hospital, Tongji Medical College, Huazhong University of Science and Technology, No.1095 Jiefang Avenue, Wuhan, 430030 China

**To the editor,**

In “Mendelian randomization and transcriptomic analysis reveal an inverse causal relationship between Alzheimer’s disease and cancer”, Zehua Dong and colleagues discovered a general protective effect of Alzheimer’s disease (AD) on cancer. However, after searching in a widely used GWAS database, IEU Open GWAS (https://gwas.mrcieu.ac.uk/) [1], we found several incorrect GWAS datasets were employed: ebi-a-GCST005921 [2] was used as exposure dataset for AD, which is actually “family history of AD”; furthermore, ukb-b-17001 and ukb-a-296 actually represent “ever had bowel cancer screening”, which were used as outcome datasets for bowel cancer (Fig. 1). Apparently, the authors used the incorrect GWAS datasets and did not explain for it. However, with a high heritability (60–80%), AD does have a strong correlation with AD family history [3]. We collated the recently published large AD GWAS dataset (ebi-a-GCST90027158) [4] and used Mendelian randomization (MR) to further investigate the relationship between AD and family history of AD (instrumental variables demonstrated in Additional file 2: Table S1, Additional file 3: Table S2). The results showed a significant bidirectional promoting causal relationship between them (Fig. 2A, Additional file 1: Figs. S1, S2). We suspected that both are driven by the same genetic variants and therefore conducted co-localization analyses in two genomic regions, including the regions near the lead SNP for ebi-a-GCST005921 and near PVRIG genetic locus (a risk gene for AD identified by the author). Within both gene regions, we discovered a very high posterior probability (100% and 99.14%) supporting Hypothesis 4 (H4), and two co-localized genetic loci (rs117310449 and rs6979218) were identified respectively (Fig. 2B, C, Table 1). Conclusively, to some extent, the family history of AD may be able to be used as a substitute for the onset of AD, but there are significant limitations that need to be discussed in the study. In addition, a large amount of GWAS datasets on AD disease have been shared in several public databases (IEU Open GWAS, GWAS Catalog), so there is no need to investigate the relationship between AD and cancer by using GWAS data on family history of AD. We suggest that the authors replace the research question in the paper with the relationship between AD family history and cancer, which is a very research-valuable question as well; and the relationship between AD and cancer needs to be further researched with the correct dataset.

**Fig. 1:**
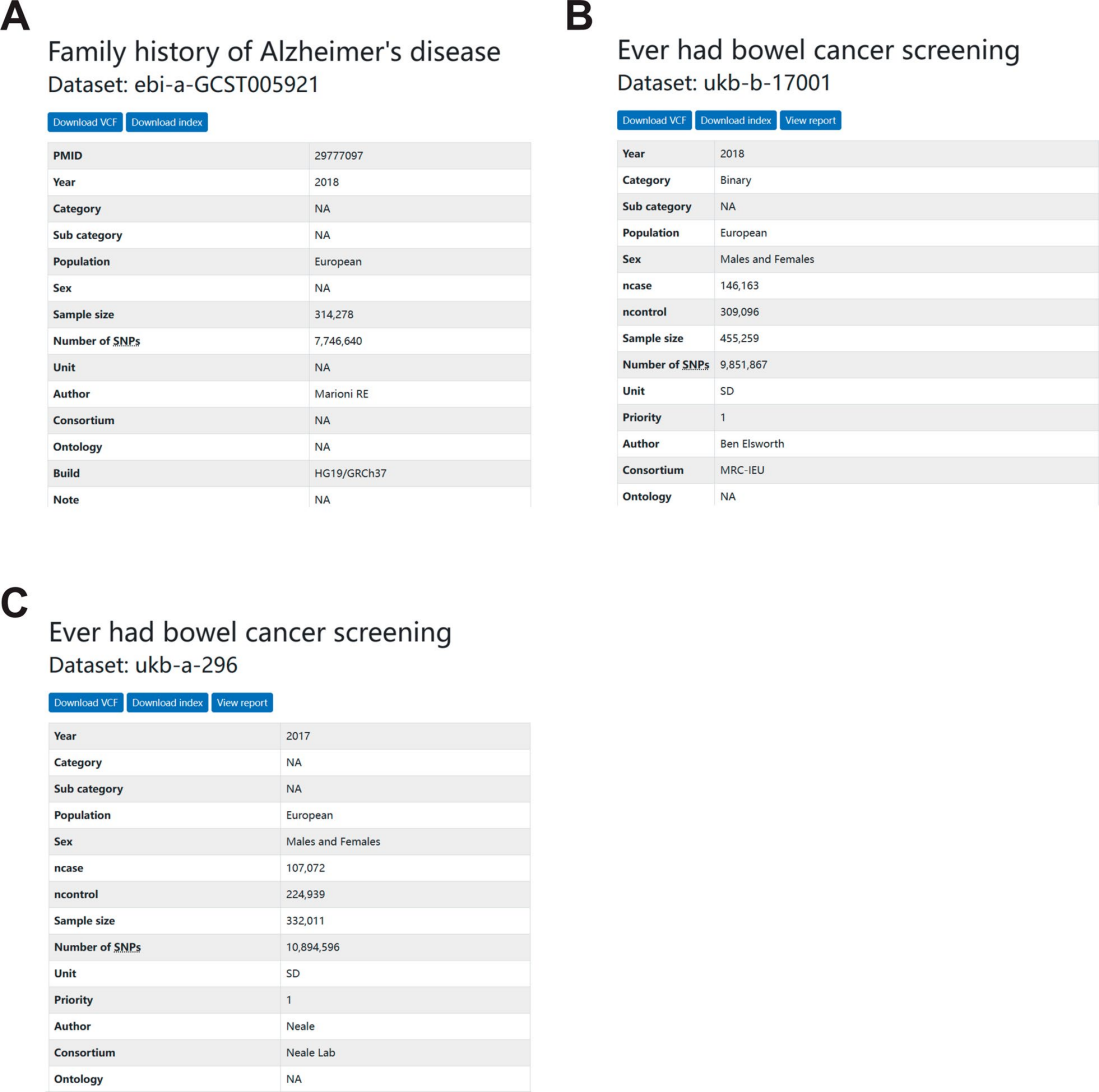
IEU Open GWAS search results for **A** ebi-a-GCST005921, **B** ukb-b-17001, and **C** ukb-a-296

**Fig. 2:**
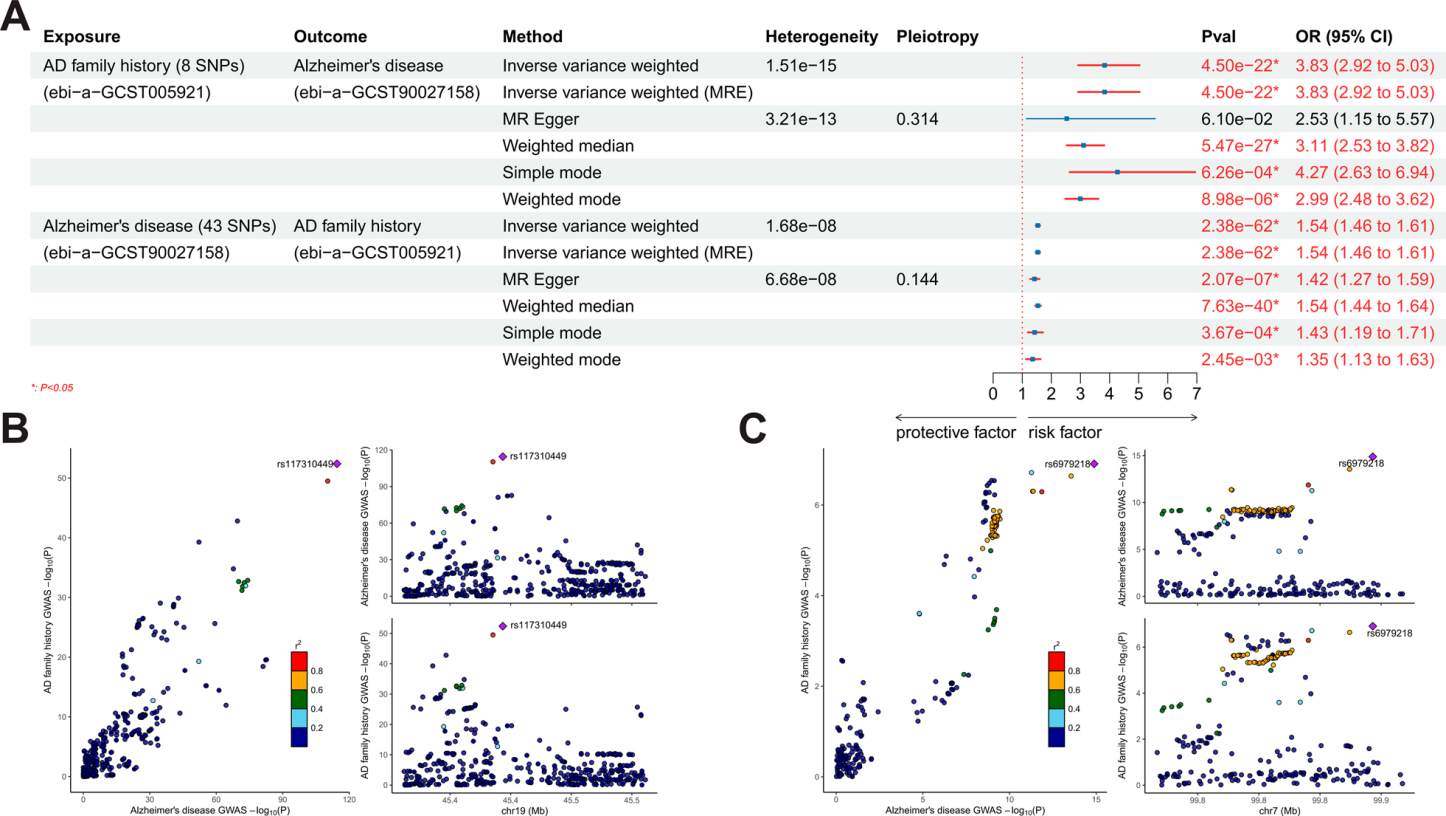
**A** Results of bidirectional Mendelian randomization between family history of AD and AD; **B** results of co-localization between AD family history and AD in the gene region near lead SNP rs429358 (± 100,000 bp) of AD family history GWAS data; **C** results of co-localization between AD family history and AD in the region near the PVRIG gene (± 100,000 bp)

**Table 1:** Results of the posterior probability obtained from co-localization analyses

Exposure	Outcome	nSNPs	Chr	Position	PP.H0.abf	PP.H1.abf	PP.H2.abf	PP.H3.abf	PP.H4.abf	Colocalized SNP
AD family history (ebi-a-GCST005921)	Alzheimer's disease (ebi-a-GCST90027158)	473	19	45411941 ± 100000 bp (around lead SNP: rs429358)	0.00%	0.00%	0.00%	0.00%	100.00%	rs117310449
		247	7	99716871–99919113 bp (around PVRIG gene loci)	0.00%	0.04%	0.00%	0.81%	99.14%	rs6979218
PVRIG (eqtl-a-ENSG00000213413)	AD family history (ebi-a-GCST005921)	301	7	99719480 ± 100000 bp (around lead SNP: rs60458236)	0.00%	0.00%	0.57%	18.97%	80.46%	rs705867
	Alzheimer's disease (ebi-a-GCST90027158)	396	7	99719480 ± 100000 bp (around lead SNP: rs60458236)	0.00%	0.00%	0.00%	28.79%	71.21%	rs55796551

“PP.H0-4.abf” represents the posterior probability supporting the H0-4 hypotheses in co-localization analysis

Additionally, the authors extracted eQTL data of brain tissue and whole blood from the GTEX database and identified PVRIG as a risk gene for AD by co-localization analysis. We believe that the robustness of the proof process in this section needs to be improved: firstly, the authors performed the analysis using the Coloc R package and the web tool Sherlock, but only reported the log Bayes factor (LBF) without the posterior probabilities of each hypothesis for the co-localization analysis; secondly, co-localization analysis is mainly adopted to evaluate whether two traits are driven by the same genetic locus, which is insufficient to establish a causal link between them [5], whereas Mendelian randomization can establish a valid causal relationship, however, the authors identified PVRIG as a risk gene for AD only after co-localization; finally, the authors used only eQTL data from GTEX without external validation, thus the conclusions remain highly limited to some extent. Therefore, collecting the cis-eQTLs near the PVRIG gene from the eQTLGen database as the exposure (instrumental variables demonstrated in Additional file 4: Table S3), we performed Mendelian randomization analyses to explore the causal relationship between PVRIG and the two AD related traits. Interestingly, the MR results showed that PVRIG was a significant protective factor for both of the AD family history and AD (Fig. 3A, Additional file 1: Figs. S3, S4), contrary to the conclusions obtained by the authors. Reverse MR analysis have ruled out the existence of a reverse causal effect (Additional file 1: Fig. S5). We recommend that the authors perform MR analyses with data from the GTEX database as well. Furthermore, we performed co-localization analyses between PVRIG and the two AD related traits in the gene region near the lead SNP for PVRIG eQTL data. The results showed that the posterior probability supporting H4 was 80.46% between PVRIG and family history of AD, and 71.21% between PVRIG and AD; the co-localized SNPs were rs705867 and rs55796551, respectively (Fig. 3B, C, Table 1).

**Fig. 3:**
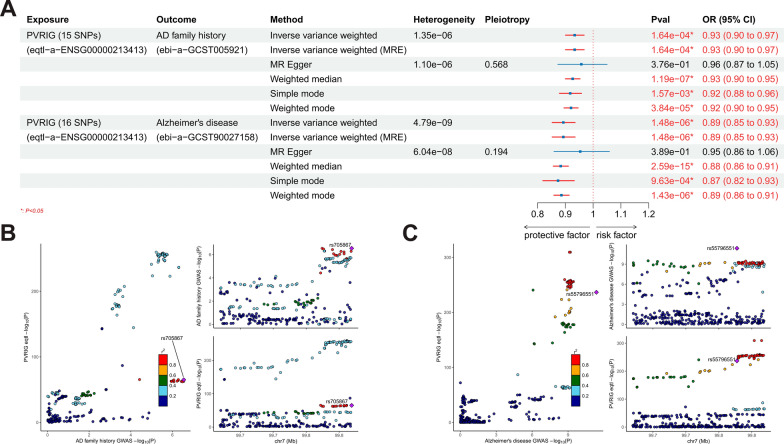
**A** Mendelian randomization results with PVRIG as exposure and family history of AD and AD as outcome; **B** results of co-localization between PVRIG and AD family history in the gene region near lead SNP rs60458236 (± 100,000 bp) of PVRIG eQTL data; **C** results of co-localization between PVRIG and AD in the gene region near lead SNP rs60458236 (± 100,000 bp) of PVRIG eQTL data

In conclusion, we identified some errors in the GWAS datasets used by the authors, which suggests that some of the conclusions have limitation and inaccuracy that require more attention; furthermore, we provided suggestions for the authors to improve analytical methodology and conducted some complementary analyses using data from other sources, which led to some opposite conclusions. Figure 4 summarized our complementary analyses. Although the conclusions of our analyses differ from part of the authors’, both of us identify a strong association between PVRIG and AD, and the cellular and molecular mechanisms between them deserve to be further investigated.

**Fig. 4:**
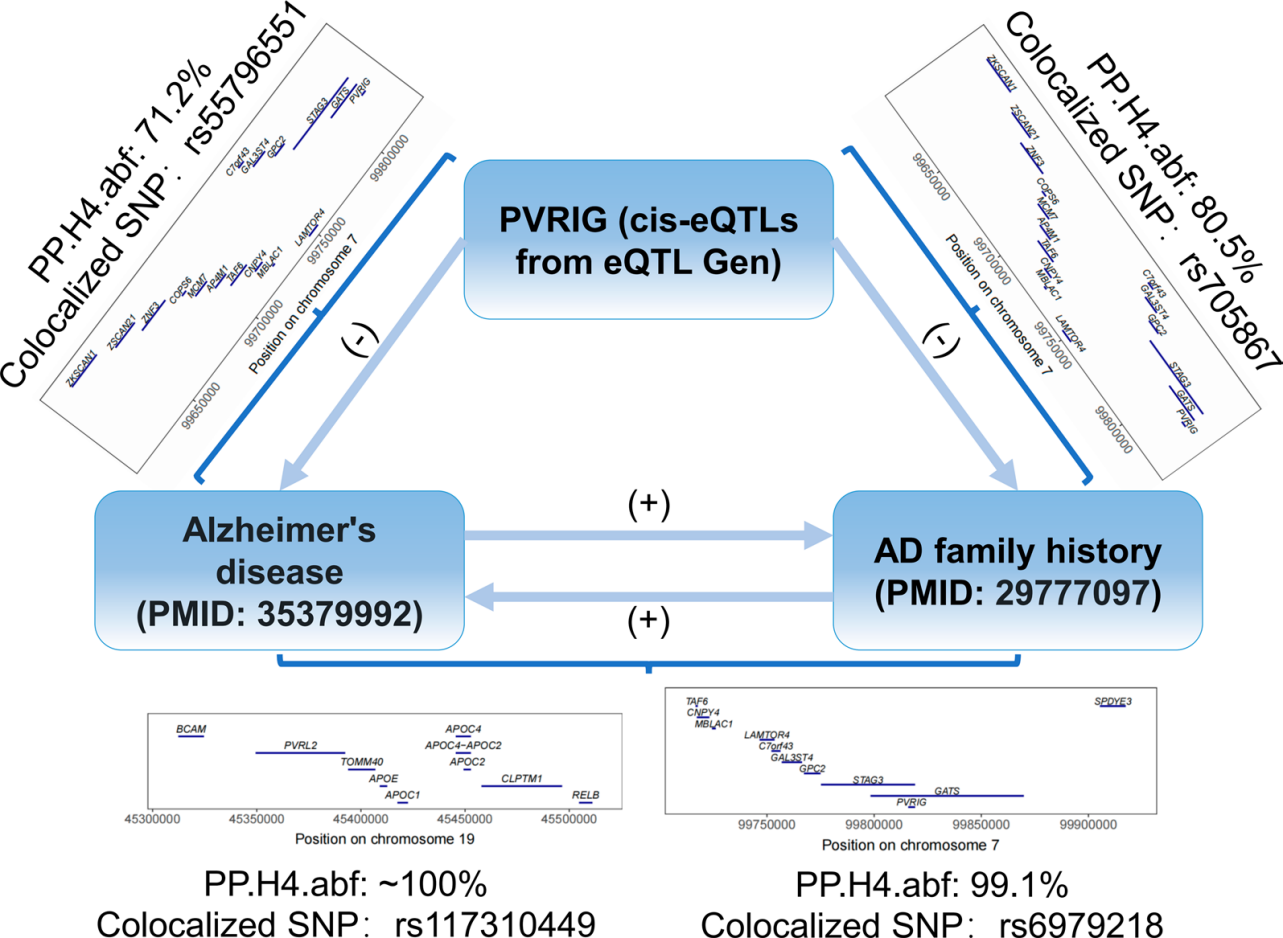
Summary of the MR analysis and the results of the co-localization analysis in this paper

### Supplementary Information

**Additional file 1: Figure S1.** Mendelian randomization with family history of AD as the exposure and AD as the outcome, this figure showed (A) scatterplot, (B) leave-one-out test plot, (C) funnel plot, and (D) forest plot, respectively. **Figure S2.** Mendelian randomization with AD as the exposure and family history of AD as the outcome, this figure showed (A) scatterplot, (B) leave-one-out test plot, (C) funnel plot, and (D) forest plot, respectively. **Figure S3.** Mendelian randomization with PVRIG as the exposure and family history of AD as the outcome, this figure showed (A) scatterplot, (B) leave-one-out test plot, (C) funnel plot, and (D) forest plot, respectively. **Figure S4.** Mendelian randomization with PVRIG as the exposure and AD as the outcome, this figure showed (A) scatterplot, (B) leave-one-out test plot, (C) funnel plot, and (D) forest plot, respectively. **Figure S5.** Mendelian randomization results with family history of AD and AD as exposure and PVRIG as outcome.

**Additional file 2: Table S1.** Instrumental variables selected from “ebi-a-GCST005921” for AD family history. Filtering condition: P < 5e−8; Clump: kb = 10000, r2 = 0.001.

**Additional file 3: Table S2.** Instrumental variables selected from “ebi-a-GCST90027158” for AD. Filtering condition: P < 5e−8; Clump: kb = 10000, r2 = 0.001.

**Additional file 4: Table S3.** Instrumental variables selected from “eqtl-a-ENSG00000213413” for PVRIG. Filtering condition: P < 5e−8; Clump: kb = 100, r2 = 0.3.

## Data Availability

The original contributions presented in the study are included in the article/supplementary material, further inquiries can be directed to the corresponding authors.
