# Supplementary material for: Identifying flaws in the GWAS datasets of a published Mendelian randomization study: complementary re-evaluation and suggestion for analytical refinements
Source: J Transl Med. 2024 Mar 26;22:311. doi: 10.1186/s12967-024-05106-w (PMC10964565; doi:10.1186/s12967-024-05106-w)
Supplement: Supplementary file 1 — Additional file 1: Figure S1. Mendelian randomization with family history of AD as the exposure and AD as the outcome, this figure showed (A) scatterplot, (B) leave-one-out test plot, (C) funnel plot, and (D) forest plot, respectively. Figure S2. Mendelian randomization with AD as the exposure and family history of AD as the outcome, this figure showed (A) scatterplot, (B) leave-one-out test plot, (C) funnel plot, and (D) forest plot, respectively. Figure S3. Mendelian randomization with PVRIG as the exposure and family history of AD as the outcome, this figure showed (A) scatterplot, (B) leave-one-out test plot, (C) funnel plot, and (D) forest plot, respectively. Figure S4. Mendelian randomization with PVRIG as the exposure and AD as the outcome, this figure showed (A) scatterplot, (B) leave-one-out test plot, (C) funnel plot, and (D) forest plot, respectively. Figure S5. Mendelian randomization results with family history of AD and AD as exposure and PVRIG as outcome. [file 12967_2024_5106_MOESM1_ESM.docx]

**Figure S1**


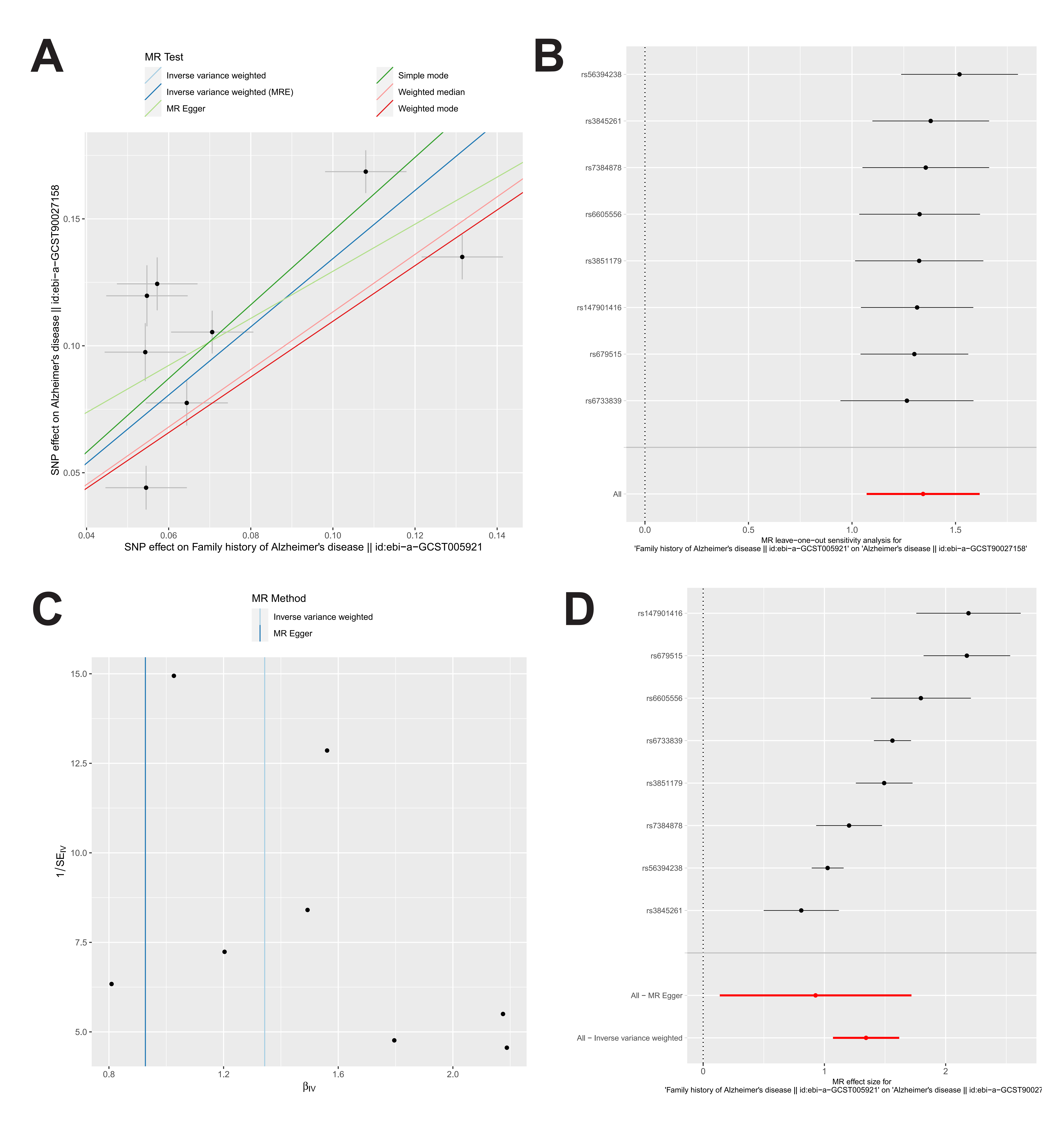


**Figure S1:** Mendelian randomization with family history of AD as the exposure and AD as the outcome, this figure showed (A) scatterplot, (B) leave-one-out test plot, (C) funnel plot, and (D) forest plot, respectively.

**Figure S2**


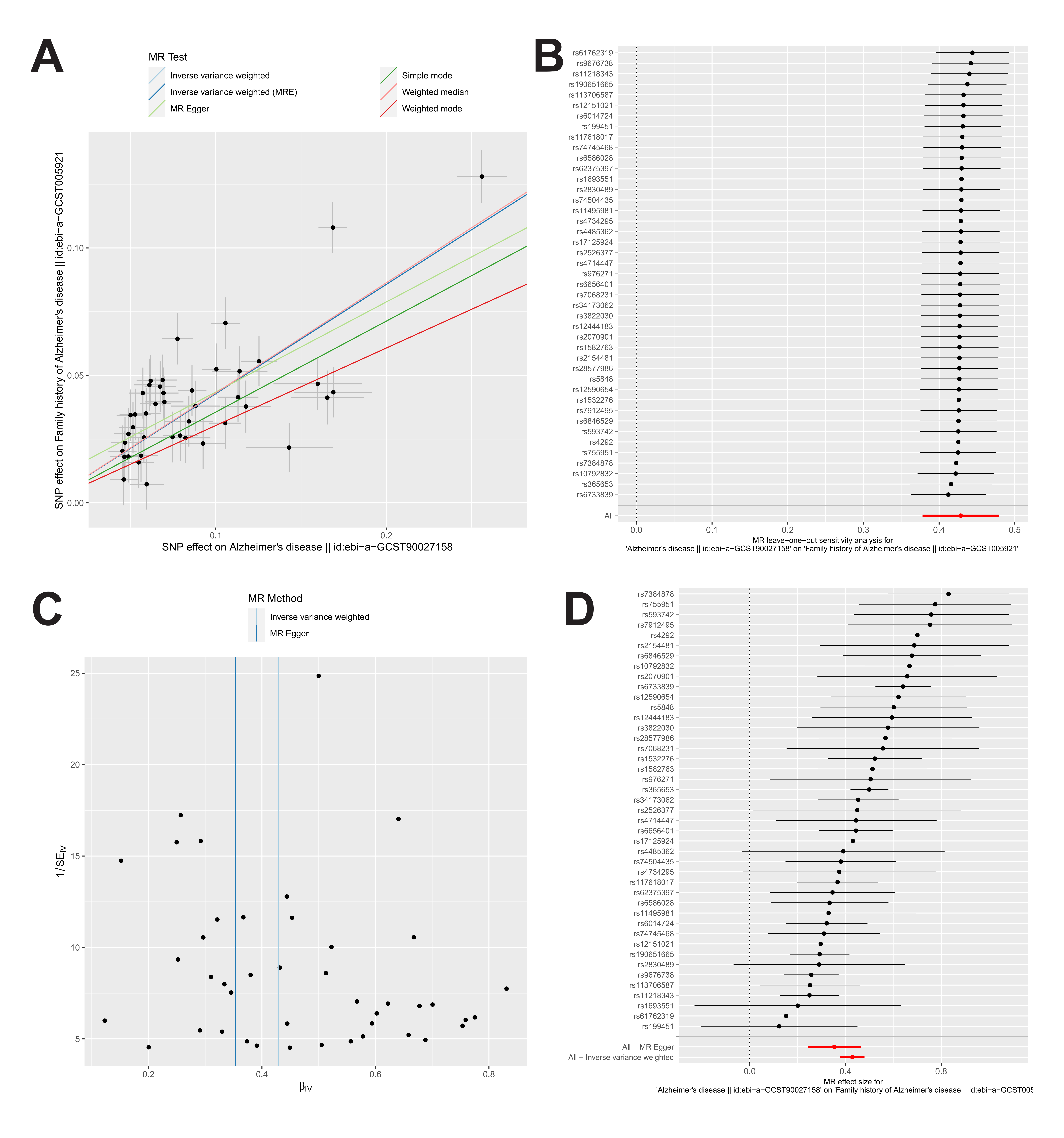


**Figure S2:** Mendelian randomization with AD as the exposure and family history of AD as the outcome, this figure showed (A) scatterplot, (B) leave-one-out test plot, (C) funnel plot, and (D) forest plot, respectively.

**Figure S3**


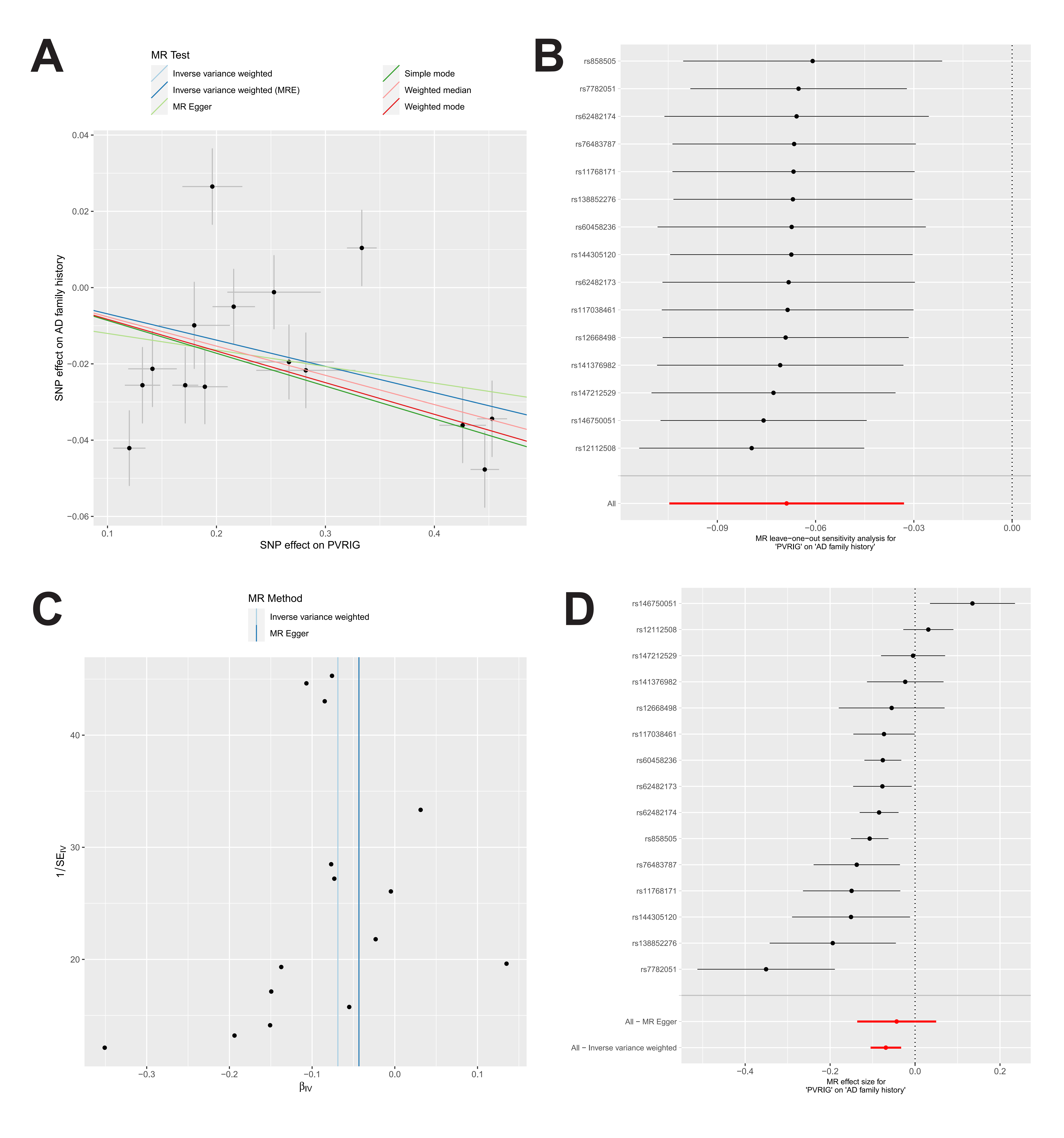


**Figure S3:** Mendelian randomization with PVRIG as the exposure and family history of AD as the outcome, this figure showed (A) scatterplot, (B) leave-one-out test plot, (C) funnel plot, and (D) forest plot, respectively.

**Figure S4**


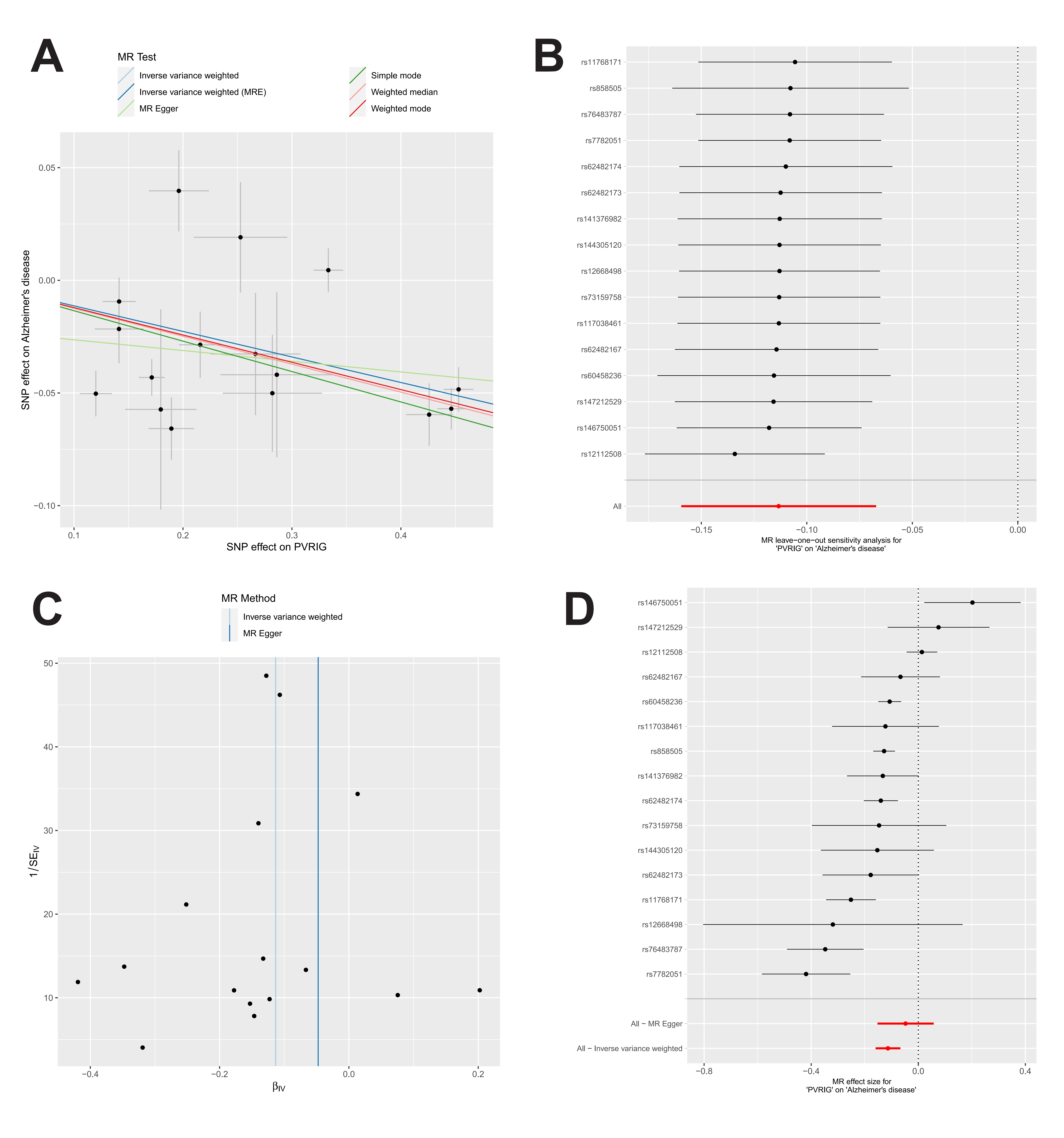


**Figure S4:** Mendelian randomization with PVRIG as the exposure and AD as the outcome, this figure showed (A) scatterplot, (B) leave-one-out test plot, (C) funnel plot, and (D) forest plot, respectively.

**Figure S5**


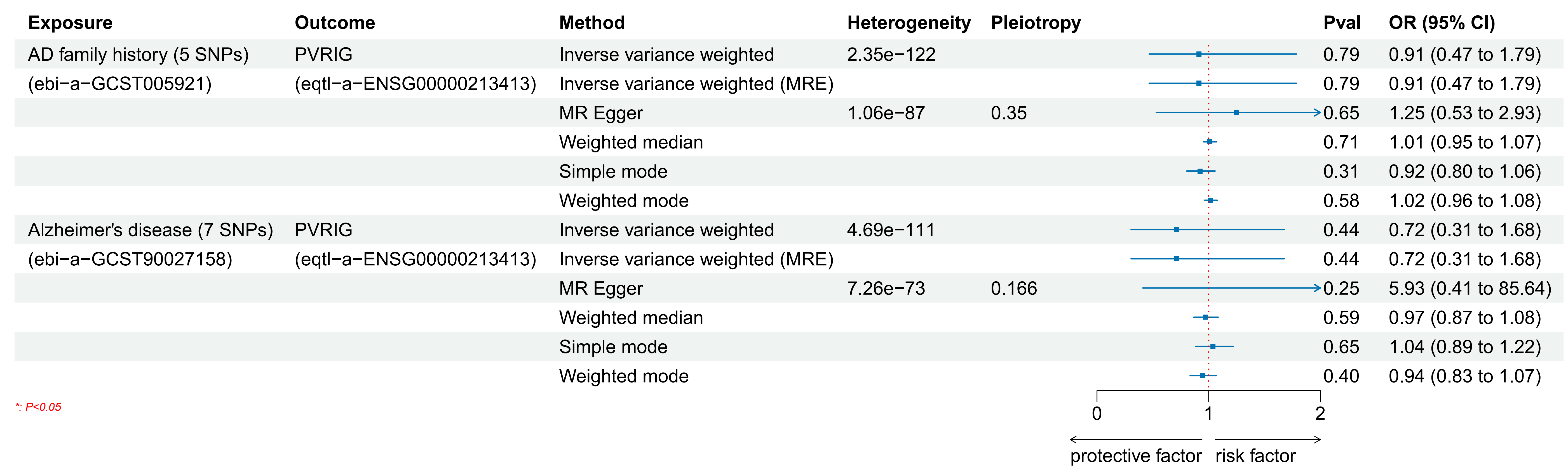


**Figure S5：**Mendelian randomization results with family history of AD and AD as exposure and PVRIG as outcome.
